# Linking human impacts to community processes in terrestrial and freshwater ecosystems

**DOI:** 10.1111/ele.14153

**Published:** 2022-12-22

**Authors:** Ian R. McFadden, Agnieszka Sendek, Morgane Brosse, Peter M. Bach, Marco Baity‐Jesi, Janine Bolliger, Kurt Bollmann, Eckehard G. Brockerhoff, Giulia Donati, Friederike Gebert, Shyamolina Ghosh, Hsi‐Cheng Ho, Imran Khaliq, J. Jelle Lever, Ivana Logar, Helen Moor, Daniel Odermatt, Loïc Pellissier, Luiz Jardim de Queiroz, Christian Rixen, Nele Schuwirth, J. Ryan Shipley, Cornelia W. Twining, Yann Vitasse, Christoph Vorburger, Mark K. L. Wong, Niklaus E. Zimmermann, Ole Seehausen, Martin M. Gossner, Blake Matthews, Catherine H. Graham, Florian Altermatt, Anita Narwani

**Affiliations:** ^1^ Swiss Federal Institute for Forest, Snow and Landscape Research (WSL) Birmensdorf Switzerland; ^2^ Institute of Terrestrial Ecosystems ETH Zürich Zurich Switzerland; ^3^ Swiss Federal Institute of Aquatic Science and Technology (Eawag) Dübendorf Switzerland; ^4^ School of Biological Sciences University of Canterbury Christchurch New Zealand; ^5^ Swiss Federal Institute of Aquatic Science and Technology (Eawag) Kastanienbaum Switzerland; ^6^ Institute of Ecology & Evolution University of Bern Bern Switzerland; ^7^ Swiss Federal Institute for Forest, Snow and Landscape Research (WSL) Davos Switzerland; ^8^ Institute of Integrative Biology, Department of Environmental Systems Science ETH Zürich Zurich Switzerland; ^9^ School of Biological Sciences The University of Western Australia Crawley WA Australia; ^10^ Department of Evolutionary Biology and Environmental Studies University of Zurich Zürich Switzerland; ^11^ Present address: Institute for Biodiversity and Ecosystem Dynamics University of Amsterdam Amsterdam The Netherlands

**Keywords:** aquatic ecology, dispersal, drift, global change, mechanism, selection, speciation, synthesis

## Abstract

Human impacts such as habitat loss, climate change and biological invasions are radically altering biodiversity, with greater effects projected into the future. Evidence suggests human impacts may differ substantially between terrestrial and freshwater ecosystems, but the reasons for these differences are poorly understood. We propose an integrative approach to explain these differences by linking impacts to four fundamental processes that structure communities: dispersal, speciation, species‐level selection and ecological drift. Our goal is to provide process‐based insights into why human impacts, and responses to impacts, may differ across ecosystem types using a mechanistic, eco‐evolutionary comparative framework. To enable these insights, we review and synthesise (i) how the four processes influence diversity and dynamics in terrestrial versus freshwater communities, specifically whether the relative importance of each process differs among ecosystems, and (ii) the pathways by which human impacts can produce divergent responses across ecosystems, due to differences in the strength of processes among ecosystems we identify. Finally, we highlight research gaps and next steps, and discuss how this approach can provide new insights for conservation. By focusing on the processes that shape diversity in communities, we aim to mechanistically link human impacts to ongoing and future changes in ecosystems.

## Graphical abstract

Community‐level responses to human impacts can differ substantially between terrestrial and freshwater ecosystems. We propose an integrative approach to explain divergent community responses by linking human impacts to the fundamental processes structuring communities (sensu Vellend, 2016): dispersal, speciation, species‐level selection and ecological drift. Using our approach combined with a literature review, we provide mechanistic insights into why responses to human impacts may differ across ecosystems and suggest ways forward to make predictions about future impacts.

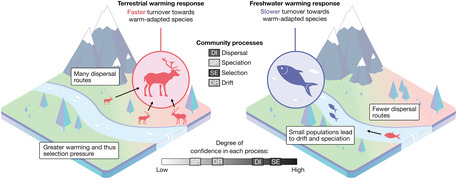

## INTRODUCTION

Humans are fundamentally altering the biodiversity and functioning of ecosystems through impacts such as habitat loss, overexploitation, climate change and biological invasions (Pörtner et al., 2021; Ruckelshaus et al., 2020). However, recent studies suggest that global change drivers may cause divergent biodiversity responses in terrestrial and aquatic communities (Blowes et al., 2019; van Klink et al., 2020), making it difficult to forecast future biodiversity changes. Indeed, a major finding of the recent IPBES Global Assessment is that differences exist in the magnitude of human impacts across ecosystems (Díaz et al., 2018; Ruckelshaus et al., 2020). One potential explanation for this finding, beyond differing impact strengths, is that the relative importance of core processes that govern biodiversity dynamics varies among ecosystems. Identifying key differences in the strength and type of processes operating in a given ecosystem is therefore a crucial step to better understand and ultimately predict human impacts.

While studies have compared differences in community processes and ecosystem properties among land and sea (Grosberg et al., 2012; May et al., 1994; Webb, 2012), comparisons are lacking for terrestrial and freshwater systems. Making these comparisons is crucial because freshwater ecosystems are intricately embedded within terrestrial habitats and linked via flows of energy, nutrients and species (Gounand et al., 2018; Soininen et al., 2015). Despite linkages, these ecosystems differ fundamentally in the physical media and landscape structure through which species interact and move. Therefore, our understanding and ability to protect terrestrial and highly threatened freshwater habitats (Belletti et al., 2020; Carpenter et al., 2011) will be greatly enhanced by studying multiple ecosystem types in a single comparative framework.

Here, we aim to build a more complete understanding of how and why responses to human impacts may vary among terrestrial and freshwater ecosystems by identifying differences and similarities in their fundamental community processes. To do this, we build on the theory of community ecology put forth by Vellend (Vellend, 2010, 2016) that distinguishes four fundamental processes which together comprehensively describe how species are gained and lost from assemblages (Box 1): dispersal, speciation, species‐level selection and ecological drift. These processes capture the mechanisms by which community attributes such as species richness, species‐abundance relationships and species turnover emerge, and are general enough to allow for comparisons across many ecosystem types. It is important to note that selection and drift here refer to community‐level processes shaping diversity in mixed‐species assemblages (Vellend, 2016), not changes in allele frequencies within populations of single species as in evolutionary biology and population genetics. Crucially, the relative importance of these community processes is likely modulated by ecosystem‐specific physical and spatial attributes, for example, properties of the media (e.g. air vs water) or geometric constraints of habitat (e.g. open vs dendritic). Thus, our goal is to develop mechanistic bridges between global change drivers and their impacts by explicitly considering both fundamental community processes and properties of the ecosystems in which they operate (Table 1).

Our approach goes beyond other recent global change frameworks, which argue for studying human impacts via the ecological scales at which they occur or the metacommunity processes with which they interact (Chase et al., 2020; Simmons et al., 2021), by explicitly considering both the ecological and evolutionary processes that mediate ecosystem impacts (Vellend, 2016). Apart from its focus on single communities, Vellend‘s theory is distinct from the related metacommunity concept because it includes the process of speciation, and all processes that affect fitness differences between species (species interactions, environmental filtering etc.) are subsumed within the process of selection (Box 1). Overall, the increased evolutionary focus via the addition of speciation greatly broadens the focal timescale when conserving ecosystems under global change, for example, when considering the long‐term recovery or extinction debt of a community.

**TABLE 1:** Key differences between freshwater and terrestrial ecosystems in physical, habitat, chemical and community properties and hypothesised effects on community processes. Note that comparisons and hypotheses are based on best available information but are not necessarily representative of every case

Category	Property	Ecosystem comparison	Relevant process	Hypothesised effect on process	References
*Physical*	Buoyancy	Water > air	Dispersal	Intrinsic dispersal ability is greater in freshwater than on land	Bonte et al., 2012; Cornell & Harrison, 2013; Srivastava & Kratina, 2013
	Terminal velocity	Air > water	Dispersal	Intrinsic dispersal ability is greater in freshwater than on land	Denny, 1990; Dawson & Hamner, 2008
	Spatial structure of environmental variation	Terrestrial > marine > freshwater	Selection (abiotic)	Greater environmental structure on land could lead to stronger species sorting compared to freshwater	Herfindal et al., 2022 (but see row below)
	Light attenuation	Water > air	Selection (abiotic)	Freshwater > terrestrial (greater niche differentiation)	Stomp et al., 2007
*Habitat*	Habitat structure	Rivers: dendritic Lakes: largely isolated habitats Terrestrial: open	Dispersal	Dispersal limitations (extrinsic) are greatest in freshwater habitats, and lowest in marine habitats	Baguette et al., 2013; Kappes et al., 2014; Wubs et al., 2016; Comte & Olden, 2018
	Habitat stability	Terrestrial > freshwater	Speciation	In situ speciation plays larger role in dynamics of freshwater than terrestrial communities	Gillespie, 2004; Miller et al., 2018
*Chemical*	Food quality	Freshwater > terrestrial	Selection (biotic)	May increase insect consumer populations and thus consumptive pressure in freshwater systems	Elser et al., 2000; Twining et al., 2021
*Community*	Species richness per area	Freshwater > terrestrial	Speciation	In freshwater habitats, the diversification rates‐per clade or lineage per unit time is highest	Miller et al., 2018; Rabosky, 2020; Miller, 2021
	Frequency of generalists	Freshwater > terrestrial	Selection (biotic)	Consumptive pressure expected to be stronger in aquatic compared to terrestrial habitats	Pringle et al., 2016; García‐Girón et al., 2020
	Naïveté to predators	Freshwater > terrestrial	Selection (biotic)	Consumptive pressure expected to be stronger in aquatic compared to terrestrial habitats	Cox & Lima, 2006; Anton et al., 2016, 2020
	Saturation of communities	Terrestrial > freshwater	Selection (biotic)	Competitive selection is weaker in freshwater habitats	Shurin et al., 2000; Shurin & Smith, 2006; Alofs & Jackson, 2014

BOX 1 **Glossary and contextualisation of the four fundamental community processes (sensu Vellend, 2016) used in the comparative approach of this paper**

*Note*: The terms selection and drift refer to community‐level processes shaping diversity in multi‐species assemblages (Vellend, 2016), and not to the changes in allele frequencies or abundances within populations of a single species studied in evolutionary biology and population genetics.

***Dispersal***: The movement of organisms among sites (Stevens et al., 2014) and the process by which species can be added to a local site from a regional species pool via immigration, or removed from a local site via emigration. Along with speciation, dispersal is one of the two processes that adds species to communities (MacArthur & Wilson, 1967; Vellend, 2016).

***Speciation***: The process by which a species splits into two reproductively isolated populations and subsequently forms two new species, either as a consequence of separation by geographic barriers (allopatric speciation) or in situ divergence (parapatric or sympatric, Coyne & Orr, 2004; Hernández‐Hernández et al., 2021). Speciation was not traditionally a focus in community ecology. However, this process is now recognised as an important mechanism influencing the size of regional species pools and the assembly of communities from them (Mittelbach & Schemske, 2015; Ricklefs, 1987). Speciation is one of the two processes that can add species to a local community, the other being immigration via dispersal.

***Selection*** (*sensu* Vellend, 2016): Species‐level selection acts on species‐specific differences in population growth rates that emerge from the fitness of all individuals in a population. The selection process thus operates on populations (as opposed to individuals in natural selection) and leads to deterministic changes in relative abundance that shape community structure (Vellend, 2010, 2016). Selection is the best‐studied of the four community processes (Cottenie, 2005), and the most diverse with respect to the ecological mechanisms it encompasses. It includes (i) the role of the environment in filtering and sorting species from the species pool (i.e. constant selection, Leibold et al., 2004; Vellend, 2010; Soininen, 2014), (ii) density and frequency‐dependent effects of interactions (e.g. competition, predation and mutualism) and (iii) impacts of environmental heterogeneity over space or time (i.e. variable selection).

***Ecological drift***: The change in relative abundances of species over time due to random variation in births and deaths of individuals (Gilbert & Levine, 2017; Hubbell, 2001; Vellend, 2010), leading to stochasticity in species' abundances over time. Drift can ultimately only erode local biodiversity due to random losses of species from communities because it does not generate or introduce new species (Vellend, 2016). Drift is likely the least well‐studied of the four community processes, despite the fact that it can play an important role in community assembly even when more deterministic processes are operating and species are not ecologically equivalent (Gilbert & Levine, 2017; Svensson et al., 2018). One signature of drift is that its influence is greater when population sizes are small, such as on islands, isolated lakes and in small habitat patches (Hubbell, 2001; Melbourne & Hastings, 2008; Orrock & Watling, 2010).

Our integrative approach building on the theory of community ecology is useful and timely because human activities impact each of the four community processes; processes which in turn generate observed responses to impacts such as species loss and turnover. Making cross‐ecosystem and process‐based comparisons using our approach will therefore help to develop a more mechanistic understanding of how humans impact biodiversity and ecosystem dynamics (Soininen et al., 2015; Twining et al., 2019). For example, knowing whether fundamental differences exist in the strength of dispersal limitation between the two ecosystems may help to understand how species track changing thermal environments during warming, or how invasive species spread. Finally, identifying differences as well as similarities in how processes operate across systems will facilitate collaboration between terrestrial and freshwater scientists who share the goal of reducing human impacts on biodiversity (Menge et al., 2009; Mokany et al., 2010).

## LINKING HUMAN IMPACTS TO COMMUNITY PROCESSES

In our approach, we ask how community responses to human impacts on terrestrial and freshwater ecosystems, such as changes in species diversity and turnover, are mediated by differences in the fundamental processes of dispersal, speciation, species‐level selection and ecological drift (Vellend, 2016). To answer this question, we first consider whether and how each of these four processes vary in strength among ecosystem types. Focusing on these processes allow us to better understand divergent responses to human impacts between terrestrial and freshwater communities because the combined actions of these processes are what that ultimately generates observed responses. Traditional approaches to studying human impacts have documented how one or several drivers, such as warming or habitat loss, alter the structure of communities and ecosystems via changes in richness, turnover and abundance. These include studies comparing terrestrial and marine ecosystems (Blowes et al., 2019) or less often terrestrial and freshwater systems (van Klink et al., 2020). While many of these studies speculate as to the ultimate causes of ecosystem responses, few explicitly consider the full set of processes shaping diversity in communities.

**FIGURE 1:**
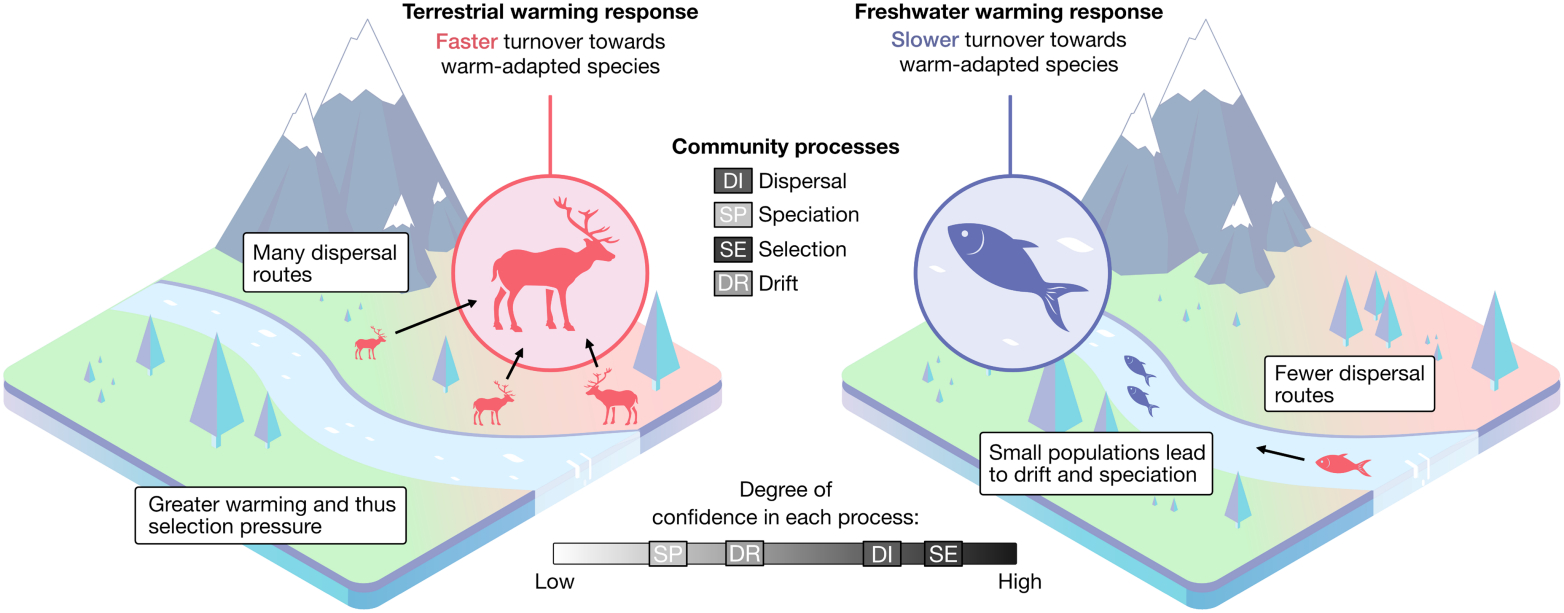
Demonstration of the impact, process and response approach for a warming scenario. Focusing on the community processes of dispersal, speciation, selection and drift can provide mechanistic insights into how humans alter the dynamics of terrestrial and freshwater systems via effects on community structure and function. We demonstrate the utility of this approach for a hypothetical climate warming scenario in which terrestrial communities (red circle) are turning over towards more warm‐adapted species (aka thermophilising) faster than freshwater communities (blue circle). Potential differences or similarities in the strength of each process among ecosystem types can then be examined to provide possible explanations for why responses may vary or not between terrestrial and freshwater habitats. In this hypothetical scenario, a pattern of slower community turnover in freshwater compared to terrestrial habitats may be explained by weaker warming impacts and greater effective dispersal limitation in freshwaters (see main text and Table 1).

To demonstrate the utility of our approach, we provide an example of how it can be used to interpret and explain a hypothetical climate warming scenario in which a freshwater community is experiencing slower turnover towards more warm‐adapted species (also known as thermophilisation) compared to a terrestrial community (Figure 1). In this scenario, the local terrestrial and freshwater communities (denoted with circles in Figure 1) are embedded within a larger landscape matrix that is experiencing warming. Here, the hypothetical community responses to warming and future recovery of assemblages are shaped by each of the four focal community processes. For example, freshwater communities may be buffered from some warming effects due to the high heat capacity of water, which slows warming. This buffering effect would decrease selection in freshwater communities for warm‐adapted species, and could drive the hypothesised pattern in which freshwater communities experience weaker warming impacts. However, the isolated nature of freshwater systems, here exemplified by the dendritic structure of a river, can reduce the ability of warm‐adapted species to enter the community via dispersal, which would slow thermophilisation.

In contrast, terrestrial systems are likely more susceptible to warming impacts due to two properties: the lower heat capacity of air compared to water, making land more easily heated, and the openness of terrestrial habitat which increases connectivity. These properties should tend to increase the number of warm‐adapted species that are selected for and that are able to disperse to the community (Figure 1). The processes of drift and speciation are also likely important drivers of change and recovery in current and future ecosystems. For example, in the longer term, some freshwater communities may recover from warming‐related species losses faster than terrestrial ecosystems through increased speciation. This could be because of the combination of ecological opportunity and relative isolation of freshwater compared to terrestrial systems, which may promote speciation (Jardim de Queiroz et al., 2022). However, any increased speciation effect would need to exceed the risk of extinction from drift, which is also high in isolated freshwater populations. By examining how these four processes together can create divergent responses and recoveries of ecosystems, we can gain a better understanding of why these differences may emerge and use this knowledge to make predictions about other systems and future impacts.

**FIGURE 2:**
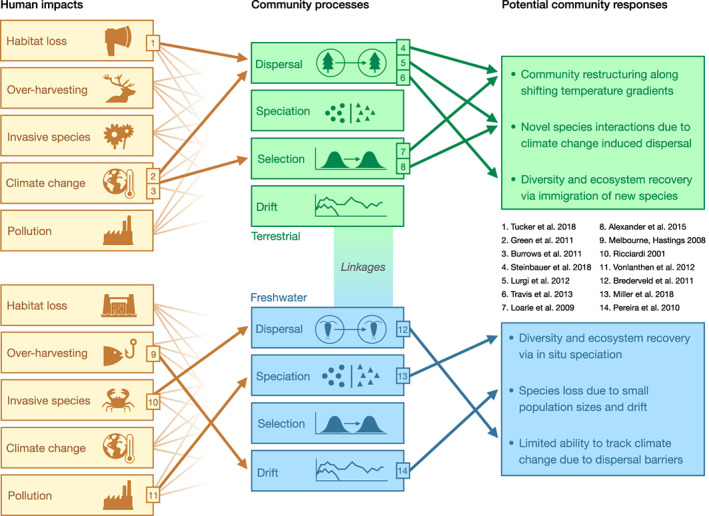
Links among impacts, processes and responses across ecosystems discussed in the text. Arrows demonstrait how human impacts can or may alter biodiversity in terrestrial and freshwater systems, via their effects on the community processes of dispersal, speciation, selection and drift. Brown arrows represent all possible effects of human impacts on individual community processes; note only a subset in bold are highlighted here. Green and blue arrows are the effects of processes of varying intensity on biodiversity responses in communities. Numbered boxes provide evidence for each highlighted link via the references listed on the right. Boxes in the rightmost column give examples of potential community responses to human impacts, as mediated by one or several community processes indicated by the arrows leading to each bullet point.

To support the implementation of our approach, we synthesise the literature to find key differences and similarities in how the four major community processes operate in terrestrial and freshwater ecosystems. We then use the differences and similarities identified to infer how terrestrial and freshwater biodiversity may respond to, and recover from, human impacts in system‐specific ways. To support these inferences, we provide representative examples of how humans impact each community process in terrestrial and freshwater ecosystems, and in turn how these processes can shape community responses to these impacts (Figure 2). Importantly, we also identify the physical, habitat, chemical and community properties that likely underlie differences in the strength of processes among ecosystems (Table 1). Although we focus primarily on the understudied comparison of terrestrial and freshwater ecosystems, we also refer to marine systems when information on oceanic environments and taxa can provide useful insights. Finally, we highlight key research areas and gaps where comparisons are missing or further research is needed.

### Dispersal

Dispersal of organisms (Box 1) may be limited by a lack of intrinsic adaptations for efficient movement or extrinsic barriers isolating species from suitable habitats. We first compare differences between terrestrial and freshwater ecosystems in species' intrinsic factors, that is, functional attributes such as differences in physiology, behaviour and life history (Comte & Olden, 2018; Stevens et al., 2014). We then compare extrinsic factors causing dispersal limitation such as geographic barriers as well as habitat configuration and connectivity (Baguette et al., 2013; Campbell Grant et al., 2007). Taken together, our literature synthesis suggests that despite the often‐strong intrinsic dispersal abilities of freshwater taxa, the extrinsic limitations of freshwater habitats lead to a lower overall dispersal potential compared to terrestrial taxa.

Studies quantifying dispersal distances for the same taxa, such as plants or invertebrates, in terrestrial and freshwater habitats suggest freshwater organisms have higher intrinsic dispersal abilities (Boedeltje et al., 2003; Kappes et al., 2014). A key factor that may shape differences in dispersal is the medium through which species move. Water is approximately 800x denser than air, and the high buoyancy this creates selects for passively dispersed life stages and promotes long‐distance dispersal by flows and currents (Bonte et al., 2012; Cornell & Harrison, 2013; Srivastava & Kratina, 2013). These differences appear to be associated with life history and morphological traits in both aquatic and terrestrial organisms (but see Green et al., 2022 for a caution on using traits as proxies for dispersal distance). For example, small‐bodied organisms generally require fewer adaptations and lower energetic costs to disperse long distances in water, whereas traits designed to increase drag are required for aerial movement (Dawson & Hamner, 2008).

Despite the intrinsic potential for long‐distance dispersal in many freshwater organisms, freshwater habitats may be effectively the most dispersal‐limited of all major ecosystems due to extrinsic factors. This is because of the high degree of spatial isolation inherent in the structure of lakes, streams and rivers (Comte & Olden, 2018) and the environmental gradients found within them (e.g. of light, Stomp et al., 2007). For example, lakes are in many ways similar to oceanic islands, in that resident species are isolated by an uninhabitable terrestrial matrix (Kappes et al., 2014). Although systems of ponds and lakes are often interconnected by a network of aquatic corridors, rivers are unsuitable habitat for many lake‐dwelling organisms (Baguette et al., 2013). Rivers are isolated both because there is little exchange of organisms across drainage basins (Leuven et al., 2009) and because dispersal is constrained by the dendritic structure and directional flow of channel networks (Campbell Grant et al., 2007; Carrara et al., 2012; Hänfling & Weetman, 2006; Wubs et al., 2016). Therefore, many riverine organisms have relatively small ranges and high levels of differentiation across rivers.

Likely due in large part to the many extrinsic barriers that are inherent in this habitat type, freshwater communities are often not saturated (Irz et al., 2004; Shurin et al., 2000), suggesting species are limited in their ability to reach new sites via dispersal (Shurin & Smith, 2006). Though studies suggest freshwater taxa may experience more extrinsic dispersal limitation on average than terrestrial taxa, isolated habitats with low connectivity also occur on land. These include oceanic and continental islands, and natural habitat islands such as serpentine soils (Harrison, 1997) and mountaintops. Overall, in both freshwater and terrestrial habitats, the interplay of intrinsic species and extrinsic landscape attributes will determine the realised dispersal distances of taxa and the larger assemblages to which they belong.

Human impacts can cause extrinsic limitations to dispersal by creating barriers, increasing heterogeneity or reducing habitat connectivity (Figure 2). In highly fragmented landscapes, distances that must be travelled in order to grow and reproduce often increase, which may lower the fitness of dispersing organisms (Stamps et al., 2005). Although effects of habitat fragmentation are most often studied in terrestrial environments, for example, tropical forests or native grasslands (Hansen et al., 2020; Stephens et al., 2008), they are thought to be more severe in freshwater systems (Fuller et al., 2015). Human‐made obstacles such as river‐crossings, culverts and dams can heavily alter species dispersal patterns, including aquatic invertebrates (Brooks et al., 2013; Sondermann et al., 2015), fish (Barbarossa et al., 2020; Duarte et al., 2021) and plants (Merritt & Wohl, 2006). In addition, in comparison with terrestrial habitats, fragmentation in dendritic river networks creates habitat patches that are smaller and more varied in size (Fagan, 2002; Fuller et al., 2015). Finally, as river networks influence dispersal between lakes, their fragmentation may also affect lake‐dwelling species (Yi et al., 2010). In response to these many impacts on dispersal in freshwaters, conservation programmes are increasingly acting to restore connectivity, for example, by moving fish upstream over dams (Harris et al., 2020).

Habitat fragmentation and landscape modification are also severe threats to terrestrial biodiversity (Fischer & Lindenmayer, 2007) and the linkages between terrestrial and freshwater ecosystems. For example, artificial constructions such as roads or fences have been shown to obstruct long‐distance dispersal in land mammals (Bartoń et al., 2019; Seidler et al., 2015; Tucker et al., 2018), plants (Dener et al., 2021) and even microbes (Le Provost et al., 2021). Interestingly, some freshwater species also utilise the terrestrial matrix for dispersal, which can help these taxa overcome effects of habitat fragmentation (Zuluaga et al., 2022). For example, freshwater invertebrates with terrestrial adult stages disperse actively over land through the air; these species better track environmental variation and are less affected by barriers such as dams (Grönroos et al., 2013; Tonkin et al., 2018). Species can also overcome an intrinsic lack of dispersal ability and extrinsic barriers by using other organisms as vectors, for example, via attachment of aquatic plants to waterfowl or ingestion of seeds by birds. Animal‐mediated dispersal not only help mitigate the effects of habitat fragmentation but can also facilitate the spread of invasive species (Coughlan et al., 2017; Incagnone et al., 2015). Dispersal by larger animals may be particularly important in freshwater systems due to lack of other ways for organisms to move among isolated patches.

### Speciation

Speciation (Box 1) is the engine generating new biodiversity, and not only many species but the process of speciation itself is under threat from ongoing human activities (Barnosky et al., 2011; Ruckelshaus et al., 2020). Studies have found that human impacts can both hinder and promote speciation, which has important implications for the long‐term recovery of ecosystem diversity (Rosenzweig, 2001). Both variation in speciation rate among lineages and the amount of time and area available for speciation to occur can influence the size of regional species pools (Miller & Román‐Palacios, 2021; Rabosky, 2020), which in turn influences the assembly of local communities. This process can also add species to communities directly via in situ speciation (e.g. Gillespie, 2004). However, despite an increasing focus on speciation as a driver of community structure, less attention has been paid to how local and regional dynamics of speciation differ among terrestrial and freshwater habitats (but see Jardim de Queiroz et al., 2022), and how these dynamics are altered by human impacts (Figure 2, Table 1). It should be noted that while the products of speciation can be quickly eradicated, recovering new species via this process often happens over much longer timescales, from centuries to millions of years depending on taxon and circumstances, and is thus outside the scope of many traditional conservation approaches.

Though few direct comparisons of speciation rates have been made between terrestrial and freshwater habitats, the highest observed diversification rates per clade or lineage per unit time occur in freshwater ecosystems, especially in lakes (Miller, 2021; Miller et al., 2018; Rabosky, 2020). This may be due in part to the greater isolation of freshwater habitats compared to terrestrial ones (Wiens, 2015). However, a recent study found that on average, terrestrial taxa have higher diversification rates than freshwater taxa, though this could be due in part to older colonisation events in freshwater (see below, Román‐Palacios et al., 2022). Future studies should compare speciation rates among terrestrial and freshwater ecosystems using matched pairs of clades with similar life histories and a range of dispersal abilities. Such studies would increase our mechanistic understanding of the relative importance of speciation among terrestrial and freshwater communities and how this process may influence responses of assemblages to human impacts.

In addition to variation in speciation rates, the amount of time and area available for speciation to occur can also influence the size of regional species pools, the rate of in situ speciation and local community diversity. Terrestrial habitats tend to be older, larger and more stable over geologic timescales than freshwater habitats such as lakes and streams (Miller, 2021), which may favour species accumulation on land at regional scales. However, because freshwater systems tend to have smaller species pools, in situ speciation may play a larger role in local diversity accumulation than in terrestrial communities, in particular for organisms that cannot disperse through air or over land (Gillespie, 2004; Miller et al., 2018). Indeed, the best‐known cases of recent rapid in situ species radiations occur in lake‐inhabiting fishes such as cichlids (McGee et al., 2020), salmoniformes (Hudson et al., 2011) and pupfish (Miller, 2021; Rabosky, 2020; Richards et al., 2021). While terrestrial radiations of comparable size do exist, such as in the Hawaiian *Drosophila* (Magnacca & Price, 2015), they have occurred over longer timescales. These differences between ecosystems in the area and time for speciation to occur have important implications for the recovery of diversity and ecological function after impacts such as habitat and species loss. For example, immigration from the larger and more connected terrestrial matrix may allow these communities to recover more quickly over shorter timescales via dispersal. In isolated freshwater communities, the much slower process of speciation may be one of the primary ways these systems recover from species loss over longer timescales.

Perhaps the primary way humans alter speciation rates, most often by decreasing them, is through the destruction and fragmentation of habitat within which new species are formed (Figure 2, Rosenzweig, 2001; Barnosky et al., 2011). On land, this occurs primarily through land‐use alteration or intensification such as deforestation and the expansion of cropland. For example, it has been estimated that approximately one‐quarter of all tree species in the Brazilian Amazon will go extinct due to habitat loss (Hubbell et al., 2008). This loss of species suggest that the future speciation potential of the Amazon will also be reduced. In freshwater systems damming, draining and eutrophication are the primary cause of habitat loss (Butchart et al., 2010; Horváth et al., 2019; Ruckelshaus et al., 2020). Due to the relatively small existing area of freshwater habitats compared to terrestrial regions and the more immediate effects on semi‐closed systems, future speciation in freshwaters may be more impacted by habitat loss as the small areas available for speciation to occur become smaller still. For example, pollution in freshwater systems can cause eutrophication and hypoxic conditions, which greatly reduce and homogenise the available amount and diversity of habitats, and often create conditions where endemic species lose all habitat in an entire system at once (Frei et al., 2022; Vonlanthen et al., 2012). Overall, human impacts on diversity may be greater in freshwater ecosystems‐ in the short term due to habitat loss and homogenisation, over medium timescales as diversity recovery via immigration is limited by dispersal barriers, and over longer timescales as the opportunity for speciation diminishes due to anthropogenic activities.

In addition to hindering speciation, human impacts may also promote speciation in terrestrial and freshwater habitats in at least two ways. First, it has been hypothesised that anthropogenic warming on land could cause once connected populations to move up in elevation into separate uplifted areas such as within a mountain range, isolating populations and potentially leading to allopatric speciation (Hua & Wiens, 2013). However, there is no evidence that this process occurs in freshwaters, as upward shifts in these habitats are associated with strong changes in environmental conditions (e.g. water velocity) that cannot be easily adapted to (Timoner et al., 2020). Second, disturbances caused by habitat homogenisation and human‐mediated dispersal can bring new species into contact and promote hybrid speciation. For example, hybrid speciation at ecological timescales in response to human‐mediated dispersal has been demonstrated in land plants (Abbott, 1992) and freshwater fish (Marques et al., 2019). Once hybrids have formed, altered ecosystems created by humans may further facilitate their survival and spread (e.g. Hoban et al., 2012). But whether this process adds new species or removes them by collapsing two parental species into one will depend on if it is associated with the gain or loss of habitat and ecological opportunity (Seehausen et al., 2008). Overall, humans have and continue to impact community diversity by reversing ongoing speciation and reducing speciation potential, but may also promote speciation in some cases.

### Selection

Understanding community responses to human impacts will require an understanding of whether and how the strength of species‐level selection (sensu Vellend, 2016, Box 1), defined as differences in mean fitness among individuals of different species, differs between ecosystems (Figure 2, Table 1). This is because human impacts such as land use, warming and invasive species alter the selection regime within communities by modifying abiotic gradients and heterogeneity (e.g. climate change velocity, Loarie et al., 2009) as well as biotic interactions such as competition, predation and mutualism (e.g. novel species interactions, Alberti, 2015; Alexander et al., 2015). Below, we compare abiotic and biotic aspects of selection on land and freshwaters, and how humans alter the selection regime through global change drivers.

Species‐level selection caused by abiotic factors may vary across ecosystems due to variation in habitat properties and the strength of environmental gradients (Table 1). For example, abiotic gradients can vary between different types of media (e.g. air vs water) due to differences in density and heat capacities as mentioned above (Table 1). In addition, it has been suggested that there is overall greater environmental structure on land than in freshwater systems (Herfindal et al., 2022). However, some gradients may be stronger in water than land, such as for light and temperature, which can promote niche differentiation in freshwater habitats (Stomp et al., 2007), especially in large and deep lakes (Seehausen & Wagner, 2014). Indeed, previous work has suggested species sorting—in which species tend to be found in sites that match their environmental preferences—is stronger on land (Govaert et al., 2021; Heino et al., 2015). For example, species sorting appears to be weakest in lakes compared to riverine and terrestrial habitats (Soininen, 2014), possibly due to the relative isolation of lakes (Heino et al., 2015). Finally, differences between ecosystems in environmental structure also have major implications for the evolution of thermal niches (Steele et al., 2018; Sunday et al., 2019) and suggest the ability of species to track thermal optima in response to climate change may differ across realms (Burrows et al., 2011).

In addition to abiotic effects, species‐level selection caused by biotic interactions may also vary between terrestrial and freshwater ecosystems (García‐Girón et al., 2020; Göthe et al., 2013; Pringle et al., 2016). Specifically, consumptive pressure via predation is thought to be stronger in aquatic systems (Alofs & Jackson, 2014; Cebrian & Lartigue, 2004; Cyr & Face, 1993). This difference may be because freshwater systems have a higher prevalence of generalist consumers, which cause strong top‐down control (Alofs & Jackson, 2014; Cyr & Face, 1993; Shurin & Smith, 2006). Alternatively, the generally higher nutritional quality of freshwater organisms (Shipley et al., 2022; Twining et al., 2019) may support larger consumer populations and thus increase predation pressure. However, the high consumptive pressure in freshwaters could also be due to a greater heterogeneity in the density of predators caused by dispersal barriers, which would lead to a higher naïveté of prey populations where predators are absent compared to terrestrial habitats (Anton et al., 2016, 2020; Cox & Lima, 2006). Finally, studies have highlighted differences in selective pressure between lakes and streams, suggesting biotic resistance to invasion is weaker in streams (Alofs & Jackson, 2014; Mitchell & Knouft, 2009). While direct comparisons of invasion pressure are generally lacking, a recent study found terrestrial systems have more invasive insects than freshwaters (Sendek et al., 2022).

As outlined above, human impacts are known to have multiple and severe effects on the selection process, to the point that they can overshadow effects of natural processes (Leprieur et al., 2008). For example, there is a large body of evidence demonstrating that climatic warming has differential impacts in terrestrial versus marine habitats; however, few studies have compared terrestrial and freshwater ecosystems. Freshwater habitats, being embedded in a terrestrial matrix, have thermal regimes that are closely tied to air. Thus, their temperatures tend to be similar to surrounding land areas, though are better buffered against extremes due to the high heat capacity of water (Figure 1, Grant et al., 2021). Among all major ecosystems, evidence suggests freshwater and terrestrial communities are less affected by warming than marine systems (Burrows et al., 2011). However, this apparent resilience may lead to accumulation of greater extinction debts compared to marine systems, which have the highest levels of assemblage turnover (Blowes et al., 2019).

An additional way humans impact species‐level selection regimes is by facilitating biological invasions. For example, it has been argued that because freshwater habitats represent a more complex matrix of interacting abiotic and biotic components, invading freshwater species can more easily affect the properties and functions of their ecosystems than terrestrial taxa (Moorhouse & Macdonald, 2015). Invasive species can also alter selection regimes in communities in a less direct way, by transmitting diseases or by altering abiotic conditions (via poisoning, bio‐fouling or changing other ecosystem properties, Blackburn et al., 2014). These processes not only affect selection regimes but can also facilitate further invasions (Green et al., 2011; Ricciardi, 2001). Invasive species effects may also be transmitted between ecosystems through various linkages, such as when invasive plants alter nutrient flows between terrestrial and freshwater habitats (Stewart et al., 2019).

### Ecological drift

Ecological drift (Box 1) is generally the most understudied of the four community processes, and has primarily been considered in microbes and terrestrial plant communities (e.g. Hubbell, 2001). However, the more general process of stochasticity is increasingly studied by ecologists (Shoemaker et al., 2020). For example, rather than being studied directly, the strength of drift has been inferred from random variation in species abundance distributions (Chase, 2010) or quantified as unexplained variation in community dynamic models (Vellend et al., 2014). Because few studies directly quantify drift (see below), in particular across several ecosystem types, we know very little about how humans impact this process in terrestrial and freshwater communities (Figure 2).

There are, however, a small number of studies quantifying drift in plant (e.g. Gilbert & Lechowicz, 2004; Gilbert & Levine, 2017; Hubbell, 2001), bacterial (Aguilar & Sommaruga, 2020; Vanwonterghem et al., 2014) and other microorganismal communities (Devercelli et al., 2016; Logares et al., 2018; Vass et al., 2020; Wu et al., 2018). Perhaps, the best‐documented studies of how drift shapes community structure come from damselfly (Odonata) communities, where species appear to closely approach ecological equivalence (McPeek & Siepielski, 2019; Svensson et al., 2018). Though more studies are needed, drift may be expected to be stronger in freshwater habitats than on land due to the much smaller total area these habitats occupy compared to their terrestrial counterparts (Wiens, 2015) and their stronger isolation. These differences may become further exacerbated by ongoing impacts such as damming and eutrophication (see below).

Despite the paucity of studies quantifying drift, it has been demonstrated that human impacts can increase the overall importance of stochastic processes, which include drift, in both freshwater and terrestrial systems. For example, species losses, nutrient addition and warming all increased the relative importance of stochastic processes in soil microbial communities (Zhang et al., 2016). Similarly, warming and nutrient addition increased the relative contribution of stochasticity, here primarily attributed to drift, among lake bacterioplankton communities (Ren et al., 2017). More generally, any anthropogenic changes which reduce community size and increase isolation (e.g. habitat fragmentation or land‐use change) should tend to increase the contribution of drift to community dynamics (Melbourne & Hastings, 2008). For example, biological invasions and over‐harvesting can cause decreased population sizes, and thereby, increase vulnerability to stochastic extinctions (Gilbert & Levine, 2013). Looking forward, a greater focus on the contribution of drift to community dynamics is warranted, as it is highly understudied but likely very important for shaping human impacts in both ecosystems.

## SYNTHESIS AND FUTURE DIRECTIONS

Here, we propose an integrative approach for comparing effects of human impacts on freshwater and terrestrial ecosystems using fundamental community processes (sensu Vellend, 2016). We find several key differences in the strength and operation of these processes that could help explain differing biodiversity responses in terrestrial and freshwater communities (Figure 2, Table S1). For example, we find evidence suggesting (i) species‐level selection in the form of species sorting due to abiotic gradients is stronger in terrestrial than in freshwater ecosystems; (ii) overall dispersal limitation may be greater in freshwater communities; but that (iii) freshwaters have especially large potential for recovery via speciation; and (iv) the biggest data gap for cross‐ecosystem comparisons is the relative influence of ecological drift. Overall, we found that quantitative comparisons across ecosystems are generally lacking, though data enabling such comparisons may be available for many organisms for the processes of dispersal and selection. In contrast, cross‐ecosystem studies of speciation rates tended to focus on a few well‐characterised organisms and such studies are largely missing for drift. Filling these gaps will be essential to fully link the processes of dispersal, speciation, selection and drift to the future dynamics and recovery of Earth's biodiversity.

In addition to filling data gaps, the mechanistic realism of our approach could be increased by considering interactions between community processes and between human impacts, as well as increased consideration of the linkages and flows between ecosystems. Interactions between community processes can modify the effect of global change drivers. This has been found in experiments and models incorporating competition and drift (Chesson, 2000; Gilbert & Levine, 2017; Orrock & Watling, 2010), and in experiments on the relative role of dispersal and selection (Ron et al., 2018). In addition, we focus primarily on the effects of single human impacts, though we recognise that these drivers interact in nature (Settele & Wiemers, 2015). For example, invasive species are often positively affected by global change, such as increases in temperature or land use intensification (Eisenhauer et al., 2012; Hellmann et al., 2008; Occhipinti‐Ambrogi, 2007). Finally, many ecosystem processes depend to some extent on linkages between systems, such as flows of nutrients and organisms between land, rivers and lakes (Soininen et al., 2015). For example, dispersal of many freshwater insects depends on adult forms that fly (Bilton et al., 2003). Furthermore, transport of nutrients and pollutants between freshwater and terrestrial systems is known to affect populations in both systems (Kraus, 2019; Kraus et al., 2021). As linkages between systems may be affected by global change drivers (Johnson et al., 2021; Kraus, 2019; Kraus et al., 2021), their role should be more fully addressed in future developments of the proposed approach.

Realising the full predictive potential of our approach will require increased research efforts and dialogue between aquatic and terrestrial ecologists, evolutionary biologists, conservationists and policymakers. Future research aimed at better forecasting human impacts across ecosystems should include targeted, quantitative studies of the strength and function of single or multiple processes in both terrestrial and freshwater ecosystems, especially on the understudied processes of speciation and drift. Furthermore, our approach could be used to parametrise a mechanistic model of impacts, processes and biodiversity outcomes. Such a model would allow researchers to make more detailed, mechanistic predictions about how diversity may change in a given community. This work would build upon recent process‐based simulation studies used to infer the mechanisms shaping diversity through deep time and across spatial scales (Hagen, Flück, et al., 2021; Hagen, Skeels, et al., 2021; Thompson et al., 2020). Mechanistic models, when parametrised with or fitted to data, can provide detailed estimates of the relative importance of processes shaping diversity across ecosystems, and increase our understanding of how biophysical properties influence the balance of processes (Table 1). Finally, the relative importance of processes in terrestrial and freshwater communities likely varies across spatial and temporal scales (Leibold et al., 2004; Levin, 1992; Simmons et al., 2021). While we discuss the temporal scale of speciation, we do not cover how other processes vary across temporal and spatial scales, which is particularly important to consider in cross‐ecosystem studies (Gounand et al., 2018). Fully addressing this question is a necessary next step to predict community response to human impacts.

Our approach highlights the need to explicitly consider the full range of processes that shape species assemblages when seeking to conserve biodiversity in terrestrial, freshwater and interconnected blue‐green landscapes. For example, ecosystem conservation efforts often focus on the connectedness and area of a given habitat without explicitly considering the underlying community processes of dispersal or drift. If the ecosystems under study are highly influenced by drift, it will be important to protect population sizes and maintain large habitat areas. If dispersal is limited due to human impacts, connectivity should be restored. In contrast, anthropogenic dispersal of species within naturally dispersal‐limited systems should be limited to reduce biological invasions. Many traditional ecosystem conservation efforts also do not take into account the effects of species‐level selection and the long‐term recovery of diversity via speciation. If selection, for example, due to warming, is a strong driver of system change, the highest priority should be to reduce the effects of this process as much as possible, for example, increase shading via habitat restoration. Finally, as speciation occurs over much slower timescales than other processes considered here, it is important to focus on preventing extinctions rather than relying on rapid speciation to recover diversity in the long term, especially in terrestrial systems.

In conclusion, to slow and potentially stop the accelerating impacts of humans on biodiversity, we must understand mechanistically how these impacts cause changes in terrestrial and freshwater ecosystems. Our process‐based approach developed here may be useful for mitigating many impacts of global change on communities for several key reasons. First, focusing on the real‐world processes that shape the diversity and structure of communities create a mechanistic bridge between a given human impact, such as climate warming, and the outcome of this process on assemblages, such as increased turnover or decreased diversity. Second, it provides a foundation for further research, especially via quantitative comparisons and mechanistic models. Thus, in the same way that these processes are meant to open the ‘black box’ of community ecology to understand community patterns (Vellend, 2010), our approach has the potential to do the same for understanding the mechanistic pathways by which humans impact Earth's biodiversity.

## AUTHOR CONTRIBUTIONS

IM, AS, AN, BM, CG and FA conceived the idea for the manuscript. IM, AS and AN reviewed the literature and wrote the first draft with substantial contributions from BM, FA, CG and MG. All authors commented on and approved the final manuscript. AN, BM, CG, FA, IL, MG and OS steered the ETH‐Domain funded BGB2020 initiative, which was the basis for this synthesis. FA, LP, CV, KB, NS, EB, MB‐J, MG, CG, BM, JB, AN, CR, DO, YV, OS and NZ secured funding to write the article.

### PEER REVIEW

The peer review history for this article is available at https://publons.com/publon/10.1111/ele.14153.

## Supporting information


Table 1.

## Data Availability

No new data were used in this study.
