# Supplementary material for: Linking human impacts to community processes in terrestrial and freshwater ecosystems
Source: Ecol Lett. 2022 Dec 22;26(2):203–18. doi: 10.1111/ele.14153 (PMC10107666; doi:10.1111/ele.14153)
Supplement: Supplementary file 1 — Table 1. [file ELE-26-203-s001.pdf]

SUPPORTING INFORMATION

Table S1 | Overview of studies comparing how human impacts may or may not differ in type or severity in terrestrial versus freshwater ecosystems, organized by the community process being impacted by humans.

Process	Human impact	Effects in freshwater ecosystems	Effects in terrestrial ecosystems
Dispersal	Habitat fragmentation	Fragmentation in freshwater systems creates smaller and less uniform patches (Fagan 2002; Fuller *et al.* 2015)	The open (non-dendritic) nature of terrestrial systems facilitates dispersal among patches (Srivastava & Kratina 2013; Fuller *et al.* 2015)
		Freshwater organisms have more intrinsic adaptations to disperse in fragmented habitats (Boedeltje *et al.* 2003)	
	Climate change		To track changing climates, species disperse and alter habitat structure and diversity (Lurgi *et al.* 2012; Travis *et al.* 2013; Steinbauer *et al.* 2018)
Speciation	Habitat loss	Because of the smaller existing area of freshwaters (Wiens 2015), negative effects of habitat destruction on speciation may be more immediate	
	Eutrophication	Eutrophication in freshwaters can cause stronger changes (e.g., depletion of oxygen) and rapid collapse of adaptive radiations through speciation reversal (Vonlanthen *et al.* 2012; Frei *et al.* 2022)	
	Climate change		Shifts in elevation could separate previously connected terrestrial populations, leading to increased speciation (Hua & Wiens 2013)
Species-level selection	Climate change	Due to buffering capacity of water, warming may be less severe in freshwater systems (Steele 1985; Vasseur & Yodzis 2004)	Selection regimes may be altered via novel competitors moving across elevation in response to climate change (Alexander *et al.* 2015)
			Increased species sorting (selection) along shifting terrestrial gradients (Loarie *et al.* 2009)
	Invasive species	Higher naïveté of prey populations to exotic predators in freshwater systems (Cox & Lima 2006; Anton *et al.* 2016, 2020)	
Ecological drift	Habitat loss	Decrease in population sizes (Pereira *et al.* 2010)	Decrease in population sizes (Pereira *et al.* 2010)
